# Betulinic Acid for Cancer Treatment and Prevention

**DOI:** 10.3390/ijms9061096

**Published:** 2008-06-27

**Authors:** Simone Fulda

**Affiliations:** University Children’s Hospital, Eythstr. 24, 89075 Ulm, Germany Tel.: +49-731 500 57034; Fax: +49-731 500 57042; E-mail: simone.fulda@uniklinik-ulm.de

**Keywords:** apoptosis, cancer, betulinic acid, mitochondria, AIF, apoptosis inducing factor, Apaf-1, Apoptotic protease activating factor-1, BA, betulinic acid, DIABLO, direct IAP Binding protein with Low PI, HtrA2, high temperature requirement protein A, IAPs, Inhibitor of Apoptosis Proteins, MOMP, mitochondrial outer membrane permeabilization, ROS, reactive oxygen species, PARP, Poly (ADP-ribose) Polymerase, Smac, second mitochondria-derived activator of caspase, TNF, tumor necrosis factor, TRAIL, tumor necrosis factor-related apoptosis-inducing ligand, zVAD.fmk, N-benzyloxycarbonyl-Val-Ala-Asp-fluoromethylketone

## Abstract

Betulinic acid is a natural product with a range of biological effects, for example potent antitumor activity. This anticancer property is linked to its ability to induce apoptotic cell death in cancer cells by triggering the mitochondrial pathway of apoptosis. In contrast to the cytotoxicity of betulinic acid against a variety of cancer types, normal cells and tissue are relatively resistant to betulinic acid, pointing to a therapeutic window. Compounds that exert a direct action on mitochondria present promising experimental cancer therapeutics, since they may trigger cell death under circumstances in which standard chemotherapeutics fail. Thus, mitochondrion-targeted agents such as betulinic acid hold great promise as a novel therapeutic strategy in the treatment of human cancers.

## 1. Introduction

Natural products have been used for combating human diseases for thousands of years, since they exhibit a wide range of biological properties that can be exploited for medical application [1]. Naturally occurring substances play an increasing role in drug discovery and development. In fact, the majority of anticancer and antiinfectious agents are of natural origin. The antitumor activity of natural products has been linked their ability to trigger cell death pathways including apoptosis in cancer cells. Apoptosis or programmed cell death is the cell’s intrinsic death program that plays a pivotal role in maintaining tissue homeostasis and that is highly conserved among different species [2]. Since apoptosis is involved in the regulation of many physiological processes, defective apoptosis signaling may lead to various pathological conditions. A hallmark of human cancers is evasion of apoptosis [3]. For example, cancer cells have the tendency to disable the mitochondrial (intrinsic) pathway of apoptosis. Besides their vital function for cellular bioenergetics mitochondria play a key role in the regulation of the point-of-no-return during apoptosis. Betulinic acid is a natural product that exhibits potent antitumor activities by triggering the mitochondrial path to apoptosis [4]. Mitochondrion-targeted agents such as betulinic acid may open new perspectives to overcome some forms of drug resistance [4].

## 2. Betulinic acid, a phytochemical with antitumor activity

**Figure 1.**
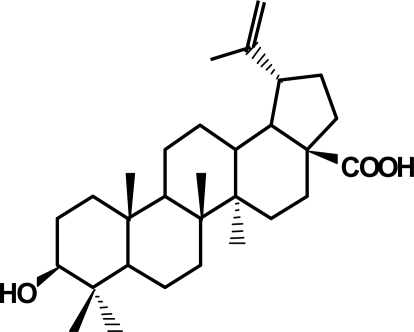
Structure of betulinic acid.

Betulinic acid (3β, hydroxy-lup-20(29)-en-28-oic acid) is a pentacyclic triterpenoid of plant origin that is widely distributed in the plant kingdom throughout the world (Figure 1) [5]. For example, considerable amounts of betulinic acid are available in the outer bark of a variety of tree species, e.g. white-barked birch trees. The reduced congener of betulinic acid, betulin (3β-lup-20(29)-en-3,28-diol), was one of the first natural products identified and isolated from plants in 1788 [6]. Betulinic acid exerts a number of biological activities. For example, betulinic acid has been shown to have antitumor properties. To this end, it is interesting to note that white birch bark (Betula alba) which contains betulinic acid, has been used by Native Americans as a folk remedy.

## 3. Mechanisms of action of betulinic acid

**Figure 2.**
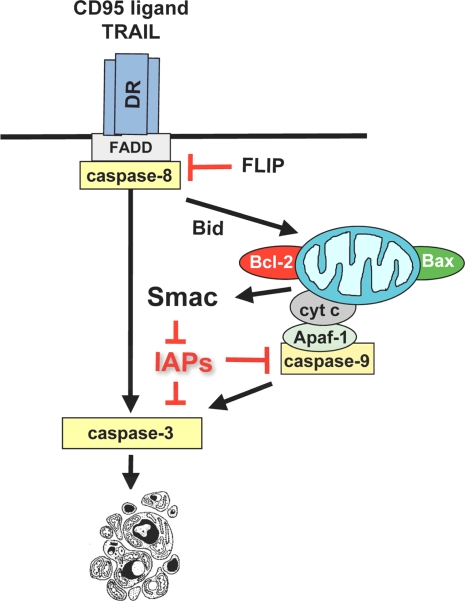
**Apoptosis pathways**. Apoptosis pathways can be initiated at the level of the mitochondria by the release of apoptogenic factors such as cytochrome c, Smac or AIF from the mitochondrial intermembrane space into the cytosol (mitochondrial or intrinsic pathway) [51]. Smac promotes apoptosis by neutralizing “Inhibitor of Apoptosis Proteins” (IAP)-mediated inhibition of caspase-3 and -9 [51]. Alternatively, apoptosis can be triggered by ligation of death receptors (DR) such as CD95 or TRAIL receptors by their cognate ligands, i.e. CD95 ligand or TRAIL (receptor or extrinsic pathway) [52]. Death receptor stimulation in turn leads to receptor trimerization, recruitment of adaptor molecules such as FADD and activation of the initiator caspase-8, which propagates the death signal to effector caspases such as caspase-3 [52–54]. The BH3 domain only protein Bid links the receptor to the mitochondrial pathway [15]: Bid is activated by caspase-8-mediated cleavage and translocates to mitochondria to promote cytochrome c release. Apoptosis can be inhibited by at various levels, e.g. by FLIP, Bcl-2 or IAPs [15, 55, 56].

Numerous studies over the last years aimed at elucidating the molecular mechanisms of betulinic acid-mediated antitumor activity. One characteristic feature of betulinic acid’s cytotoxicity is its ability to trigger the mitochondrial pathway of apoptosis in cancer cells. Apoptosis is an intrinsic program of cell death that is present in every cell and regulated by defined signaling pathways (Figure 2).

### 3.1. Activation of the mitochondrial pathway by anticancer therapeutics

The intrinsic (mitochondrial) pathway of apoptosis is triggered upon treatment with chemotherapeutic agents or upon radiotherapy as the result of a DNA damage or cellular stress response [7]. A pivotal initial step in the activation of the mitochondrial pathway is the permeabilization of the outer mitochondrial membrane [7]. During this process, both the mitochondrial outer and inner membranes are permeabilized, which in turn results in the release of soluble proteins from the mitochondrial interspace into the cytosol, for example cytochrome c, Smac or AIF [7]. A long list of protein factors and second messengers have been identified that can positively or negatively regulate permeabilization of the outer mitochondrial membrane [8]. Thus, factors that can directly induce mitochondrial outer membrane permeabilization can act as effective cytotoxic agents.

### 3.2. Induction of mitochondrial outer membrane permeabilization by betulinic acid

Betulinic acid has been reported to induce apoptosis via direct mitochondrial perturbations. When added to isolated mitochondria in cell-free systems, betulinic acid induced loss of mitochondrial membrane potential in a manner that was not affected by the caspase inhibitor zVAD.fmk and yet was inhibited by bongkrekic acid, an inhibitor of the permeability transition pore complex [9]. Also in intact cells, betulinic acid was shown to trigger cytochrome c in a caspase-independent and permeability transition pore-dependent manner [10]. In a cell-free system comprising mitochondria, cytosols, and purified nuclei, mitochondria undergoing betulinic acid-induced permeability transition mediated cytosolic caspase activation and nuclear fragmentation via the release of soluble factors, such as cytochrome c or AIF [9]. Antiapoptotic Bcl-2 family proteins such as Bcl-2 and Bcl-X_L_ inhibited all mitochondrial and cellular manifestations of apoptosis induced by betulinic acid, as did bongkrekic acid [9], indicating that mitochondrial permeability transition is required for these events. Perturbance of mitochondrial function constitutes a central coordinating event in betulinic acid-induced apoptosis leading to caspase activation and apoptotic DNA fragmentation. Mitochondria from cells, which were treated with betulinic acid, induced cleavage of both caspase-8 and caspase-3 in cytosolic extracts. Cytochrome c, released from mitochondria undergoing betulinic acid-mediated permeability transition, activated caspase-3 but not caspase-8 in a cell-free system. Cleavage of caspase-3 and -8 was preceded by disturbance of mitochondrial membrane potential and by generation of reactive oxygen species. In addition, activation of caspases was restricted to cells that already had lost their mitochondrial membrane potential further suggesting that mitochondrial alterations were involved in betulinic acid-induced activation of caspases. Overexpression of Bcl-2 and Bcl- X_L_ conferred resistance to betulinic acid at the level of mitochondrial dysfunction, protease activation, and nuclear fragmentation indicating that these events occurred downstream of the Bcl-2- or Bcl-X_L_-controlled checkpoint of apoptosis. These findings suggest that caspase-8 is activated downstream of mitochondria during betulinic acid-induced apoptosis. Activation of the caspase cascade was required for betulinic acid-triggered apoptosis, as broad-spectrum peptide inhibitors of caspases completely abrogated betulinic acid-triggered apoptosis. Interestingly, neuroblastoma cells resistant to doxorubicin-mediated apoptosis were still responsive to treatment with betulinic acid [11]. This suggests that betulinic acid may overcome some forms of drug resistance.

Generation of reactive oxygen species (ROS) upon treatment with betulinic acid has been reported to be involved in initiating mitochondrial membrane permeabilization. To this end, ROS generation was detected in cancer cell lines of different origin that were treated with betulinic acid [12–14]. Incubation with antioxidants prior to adminstration of betulinic acid rescued cells from undergoing apoptosis suggesting that ROS production was involved in mediating cell death. Also, ROS generation was linked to activation of pro-apoptotic p38 and SAP/JNK kinases with no change in the phosphorylation of ERK indicating that ROS act upstream of the MAPKs in the signaling pathway of betulinic acid [13].

### 3.3. Regulation of betulinic acid-induced apoptosis by Bcl-2 family proteins

Proteins of the Bcl-2 family are among the many signal transduction proteins that can act on mitochondria to regulate outer membrane permabilization. Bcl-2 family proteins comprise both anti-apoptotic members, e.g. Bcl-2, Bcl-X_L_, Mcl-1, as well as pro-apoptotic molecules such as Bax, Bak, Bad and BH3 domain only molecules [15]. Imbalances in the ratio of anti-apoptotic versus pro-apoptotic Bcl-2 proteins may tip the balance in favor of tumor cell survival instead of cell death [15].

Betulinic acid has been reported to modulate expression levels of different Bcl-2 family proteins. For example, treatment with betulinic acid resulted in upregulation of the pro-apoptotic Bcl-2 family protein Bax in neuroblastoma, glioblastoma and melanoma cells, whereas Bcl-X_S_ was found at elevated levels in betulinic acid-treated neuroblastoma cells [12, 14, 16]. Expression levels of pro-apoptotic proteins Bak and Bad were not altered in response to betulinic acid in melanoma cells [16, 17]. While expression levels of anti-apoptotic Bcl-2 remained unchanged upon incubation with betulinic acid in neuroblastoma and squamous cell carcinoma cells, an increase in Bcl-2 protein levels was reported in glioblastoma cells [12, 14, 18]. Also, betulinic acid triggered upregulation of Mcl-1, another anti-apoptotic Bcl-2 family protein, in melanoma cells, whereas no changes in Mcl-1 levels were detected in squamous cell carcinoma cells [16–18]. As fas as Bcl-X_L_ is concerned, no alterations in expression levels were reported upon exposure to betulinic acid in neuroblastoma, glioblastoma or melanoma cells [12, 14, 17]. These findings suggest that betulinic acid regulates Bcl-2 family proteins in a context-dependant manner.

Moreover, betulinic acid has been reported to induce apoptosis in a p53- and CD95-independent manner. To this end, apoptosis upon treatment with betulinic acid was not associated with accumulation of wild-type p53 protein [12, 14, 16, 19–21]. Also, betulinic acid similarly induced apoptosis in p53 mutant and p53 wild-type cell lines and was also active in p53 deficient melanoma cells [16, 22]. Moreover, betulinic acid triggered apoptosis independent of CD95-ligand/receptor interaction [12, 14, 23].

### 3.4. Modulation of NF-κB activity by betulinic acid

Betulinic acid has also been reported to modulate activity of the transcription factor nuclear factor-κB (NF-κB), a key regulator of stress-induced transcriptional activation. Betulinic acid was identified as a potent activator of NF-κB in a number of cancer cell lines [24]. Betulinic acid-induced NF-κB activation involved increased IKK activity, phosphorylation of IκBα at serine 32/36 followed by degradation of IκBα and nuclear translocation of the NF-κB subunit p65. Reporter assays confirmed that NF-κB that was activated by betulinic acid is transcriptionally active. Interestingly, inhibition of betulinic acid-induced NF-κB activation by different chemical inhibitors (proteasome inhibitor, antioxidant, IKK inhibitor) also impaired betulinic acid-induced apoptosis. Importantly, specific NF-κB inhibition by transient or stable expression of IκBα super-repressor inhibited betulinic acid-induced apoptosis in some neuroblastoma cells, while transient expression of IκBα super-repressor had no influence on betulinic acid-induced apoptosis in other cell lines. These findings indicate that activation of NF-κB by betulinic acid promotes betulinic acid-induced apoptosis in a cell type-specific manner. By comparison, betulinic acid was shown to interfere with NF-κB activation and NF-κB-regulated gene expression triggered by carcinogens and inflammatory stimuli [25]. These findings may provide a molecular basis for the ability of betulinic acid to suppress inflammation and modulate the immune response. Together, these findings point to a context-dependant function of NF-κB in the regulation of betulinic acid-mediated apoptosis.

## 4. Additional anticancer effects of betulinic acid

Betulinic acid has also been reported to inhibit aminopeptidase N, an enzyme that is involved in the regulation of angiogenesis and overexpressed in several cancers [26–28]. In addition, betulinic acid was reported to exert antiangiogenic effects by inhibiting growth factor-induced *in vitro* angiogenesis in endothelial cells, possibly by affecting mitochondrial functions [26]. Further, the antiangiogenic activity of betulinic acid was attributed to activation of selective proteasome-dependent degradation of the transcription factors specificity protein 1 (Sp1), Sp3, and Sp4, which regulate vascular endothelial growth (VEGF) expression [29]. Compared to betulinic acid, 20,29-dihydro-betulinic acid derivatives were claimed to posses better anti-angiogenic properties as betulinic acid [30]. Also, betulinic acid was shown to inhibit the catalytic activity of topoisomerase I [31]. Furthermore, betulinic acid exerts context-dependant effects on the cell cycle. While betulinic acid was found to reduce expression of p21 protein in melanoma cells, an increase of p21 protein was observed upon treatment with betulinic acid in glioblastoma cells [12, 32]. Alterations in cell cycle progression in response to betulinic acid were also highly dependant on individual cell lines [32]. Whether betulinic acid-mediated cell cycle changes are linked to its antitumor activity remains to be addressed in future studies.

## 5. Anticancer activity of betulinic acid

The antitumor cytotoxicity of betulinic acid has been extensively studied in a panel of cancer cell lines, primary tumor samples and xenograft mouse models (Table 1). While initial reports suggested that betulinic acid is selectively cytotoxic against melanoma cell lines [33], anticancer activity was subsequently also reported against other types of human cancers including neuroblastoma, glioblastoma, medulloblastoma, Ewing tumor, leukemia as well as several carcinoma, i.e. head and neck, colon, breast, hepatocellular, lung, prostate, renal cell, ovarian or cervix carcinoma [12, 14, 18, 22, 23, 34–39]. In addition to tumor cell lines, Betulinic acid was also cytotoxic against primary cancer cells isolated from tumor specimens obtained from neuroblastoma, glioblastoma and leukemia [14, 23, 38, 39]. Also, betulinic acid was cytotoxic in different models of drug resistance, for example primary pediatric acute leukemia samples that were refractory to standard chemotherapeutic agents [14, 23]. Thus, betulinic acid may overcome certain forms of drug resistance. Further, there is evidence that betulinic acid is preferentially cytotoxicity against metastatic over non-metastatic melanoma cell lines [32]. Moreover, betulinic acid cooperated with different cytotoxic stimuli to suppress tumor growth, including ionizing radiation [16], chemotherapeutic drugs [40] [41] or the death recpetor ligand TRAIL [42]. This suggests that betulinic acid may be used as sensitizer in combination regimens to enhance the efficacy of anticancer therapy. By comparison, normal cells of different origin have been reported to be much more resistant to betulinic acid than cancer cells pointing to some tumor selectivity [16, 22, 38, 39].

Besides its potent antitumor activity *in vitro*, betulinic acid also suppressed tumor growth in several animal models of human cancer. In a xenograft mouse model of ovarian cancer administration of betulinic acid significantly increased the survival time [22]. Also, betulinic acid suppressed tumor growth in a melanoma xenograft model [33]. Also *in vivo*, betulinic acid cooperated with chemotherapeutic agents such as vincristin to reduce lung metastasis in a metastatic melanoma model [41]. Of note, no systemic toxicities or weights loss were observed in betulinic acid-treated mice even at high systemic doses of betulinic acid [22, 33]. Pharmacokinetic studies in mice bearing melanoma xenografts demonstrated that betulinic acid was well absorbed and distributed with highest concentrations found within the tumor [43, 44]. Phase I/II studies of 3-o-(3′,3′-dimethylsuccinyl) betulinic acid (bevirimat) in patients with human immunodeficiency virus (HIV) infection demonstrated that single oral doses of bevirimat were well tolerated and that plasma concentrations ranged from 8 to 58 μg/ml [45, 46]. This indicates that plasma levels of betulinic acid could be achieved after oral administration in humans that correspond to concentrations, which were found to exert antitumor activity *in vitro*.

Furthermore, betulinic acid was reported to harbor anticarcinogenic properties that could be exploited in cancer prevention settings. To this end, betulinic acid was shown already more than a decade ago to inhibit tumor formation in mouse skin two-stage carcinogenesis [47]. Betulinic acid is currently under evaluation as a topical agent in a phase I/II clinical trial for the treatment of dysplastic nevi with the potential to transform into melanoma.

In addition to betulinic acid, a variety of betulinic acid derivatives were developed with the aim to increase the anticancer potency and to improve the pharmacokinetic properties. For example, replacing the cyano group with a methoxycarbonyl was reported to markedly enhance the apoptosis-inducing activities of betulinic acid [48]. In addition, these new BA analogues showed higher plasma and tissue levels compared to betulinic acid [48]. Further, C-3 modified Betulinic acid derivatives proved to have better *in vivo* anti-tumor efficacy as compared to betulinic acid *in vivo* against human colon cancer and also displayed favorable pharmacokinetic properties [49]. Moreover, 17-carboxylic acid modified 23-hydroxy betulinic acid ester derivatives demonstrated for cytotoxic activity on five cancer cell lines *in vitro*: all tested compounds showed higher cytotoxic activity as compared to 23-hydroxy betulinic acid and betulinic acid *in vitro* and also *in vivo* [50].

## 6. Conclusions

The natural compound betulinic acid shows potent anticancer activity through activation of the mitochondrial pathway of apoptosis in cancer cells. Betulinic acid may also be used in combination protocols to enhance its antitumor activity, for example with chemo- or radiotherapy or with the death receptor ligand TRAIL. Because of its relative selective cytotoxicity against malignant compared to normal cells, betulinic acid is a promising new experimental anticancer agents for the treatment of human cancers.

## Figures and Tables

**Table 1.** *In vitro* cytotoxic effect of betulinic acid on human cancer cell lines.

Cancer type	ED50 (μg/ml)	References
melanoma	1.1–4.8	[33]
neuroblastoma	2–10	[14]
medulloblastoma	3–15	[39]
glioblastoma	5–16	[39]
head & neck cancer	8	[18]
ovarian carcinoma	1.8–4.5	[22]
cervix carcinoma	1.8	[22]
lung carcinoma	1.5–4.2	[22]
leukemia	2–15	[23]
